# Supplementary material for: AlphaFold prediction of structural ensembles of disordered proteins
Source: Nat Commun. 2025 Feb 14;16:1632. doi: 10.1038/s41467-025-56572-9 (PMC11829000; doi:10.1038/s41467-025-56572-9)
Supplement: Supplementary file 1 — Supplementary Information [file 41467_2025_56572_MOESM1_ESM.pdf]

SUPPLEMENTARY INFORMATION

AlphaFold Prediction of Structural Ensembles of Disordered Proteins

Z. Faidon Brotzakis[1,2,†], Shengyu Zhang[1,†], Mhd Hussein Murtada[1,†]
and Michele Vendruscolo[1,*]

[1]*Centre for Misfolding Diseases, Yusuf Hamied Department of Chemistry, University of Cambridge, Cambridge, UK*
[2]*Institute for Bioinnovation, Biomedical Sciences Research Center "Alexander Fleming", 16672 Vari, Greece*

† Equal contributions
* Correspondence: mv245@cam.ac.uk

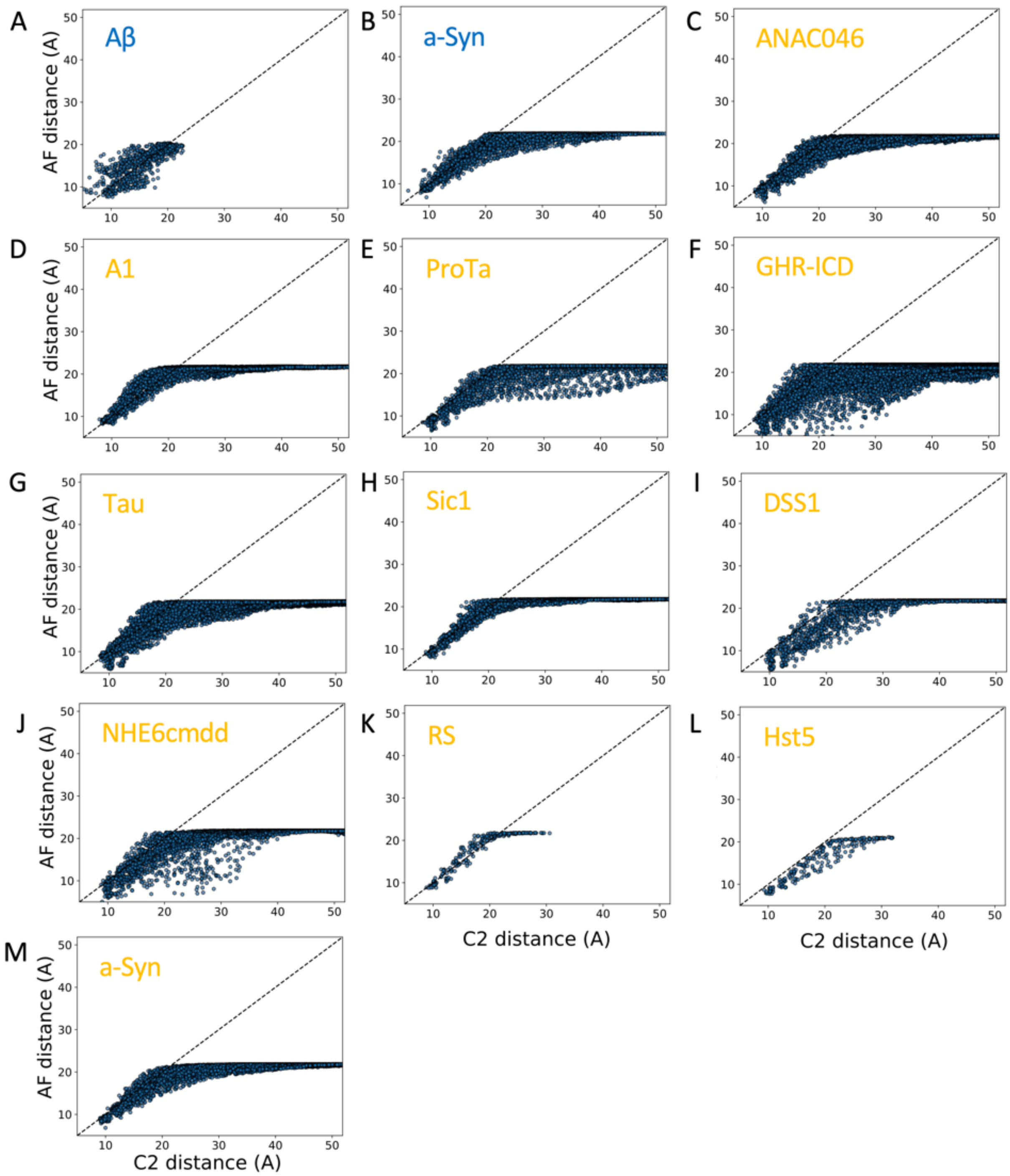

Supplementary Figure 1. Comparison of average inter-residue distances predicted by AlphaFold and back-calculated from molecular simulations. (A,B) Distances from all-atom molecular dynamics (MD) simulations. **(C-M)** Distances from CALVADOS-2 (C2) simulations.

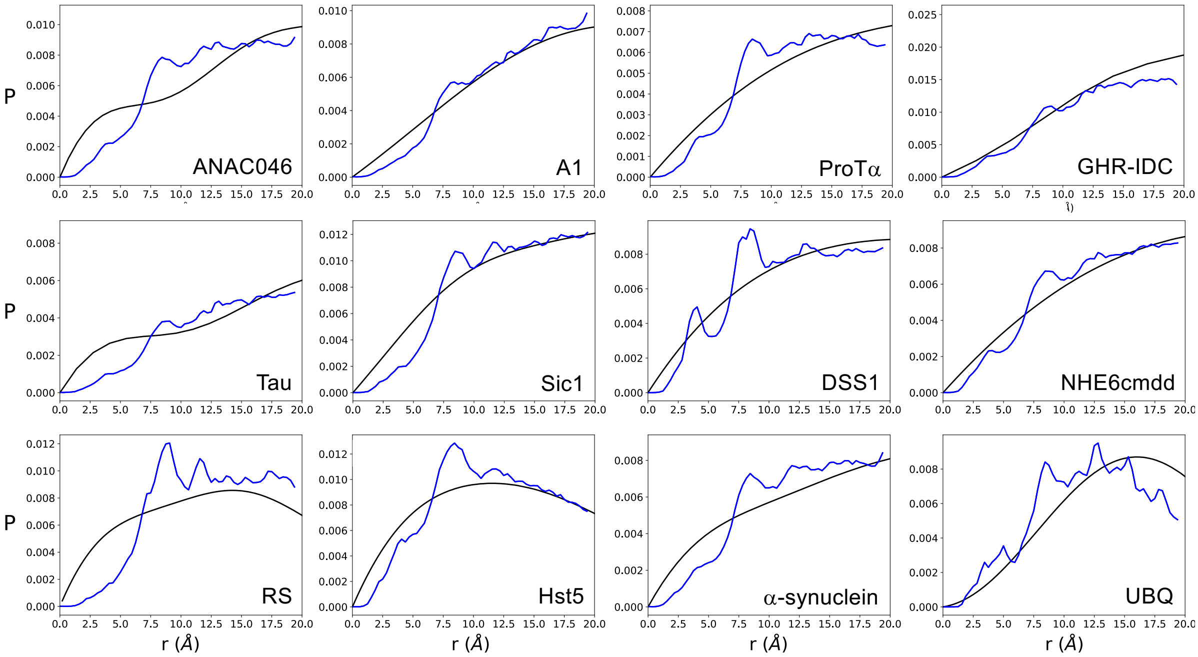

Supplementary Figure 2. Comparison of inter-residue distance distributions obtained by SAXS and predicted by AlphaFold. We used a set of 11 proteins for which both SAXS and NMR diffusion measurements were available[1]. SAXS-derived inter-residue distance distributions are shown in black, and AlphaFold-predicted average inter-residue distance distributions are shown in blue. The cut-off distance in the AlphaFold predictions is 21.84 Å.

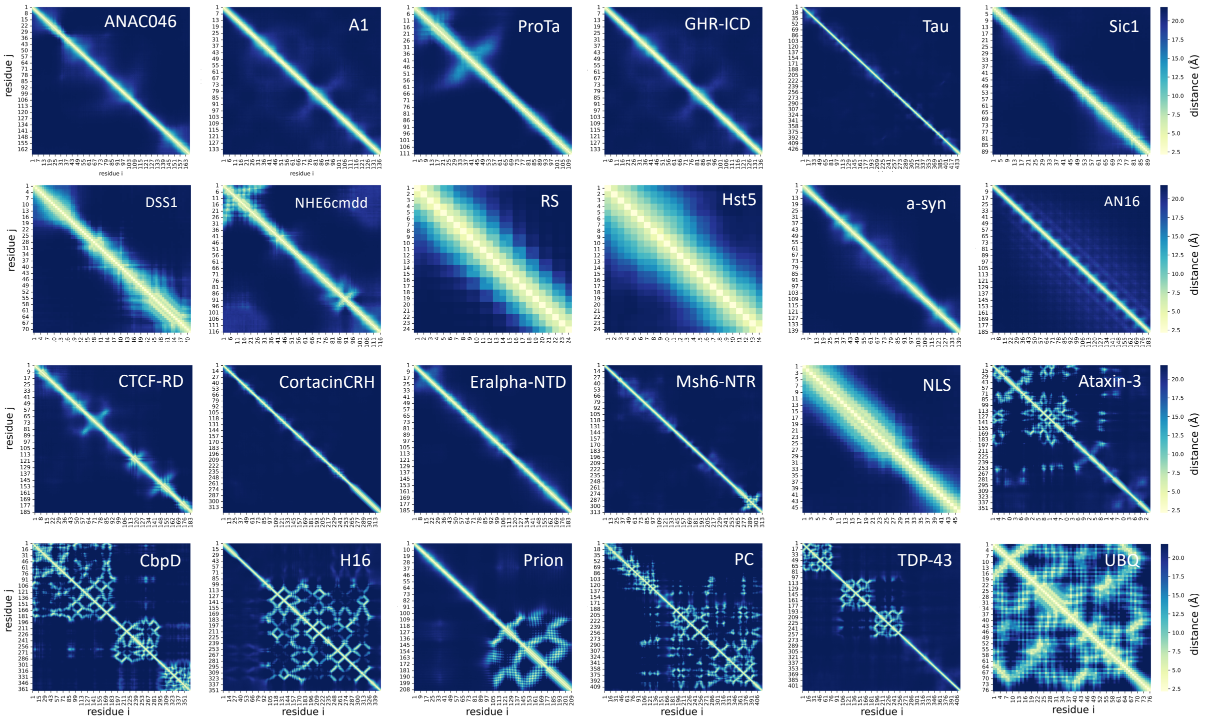

Supplementary Figure 3. AlphaFold distograms of the proteins analyzed in this work. The distograms report the average inter-residue distances predicted by AlphaFold for the intrinsically disordered proteins, partially disordered proteins and folded proteins that we reported in this work.

A

Protein	AF-MI	AF	Exp. Rg (ATSAS) [1]	Length	v
ANAC046	3.63	3.16	3.6 ± 0.3	167	0.55
A1	2.65	2.72	2.5 ± 0.1	137	0.49
ProTa	4.14	3.03	3.7 ± 0.2	111	0.62
GHR-ICD	6.4	3.84	6.0 ± 0.5	351	0.56
Tau	7.18	4.71	6.4 ± 0.5	441	0.54
Sic1	2.85	2.8	2.9 ± 0.1	90	0.58
DSS1	2.6	2.06	2.5 ± 0.1	71	0.58
NHE6cmdd	3.12	2.74	3.2 ± 0.2	116	0.57
RS	1.34	1.72	1.26 ± 0.08	24	0.57
Hst5	1.36	1.57	1.34 ± 0.05	24	0.58
a-syn	3.64	2.93	3.56 ± 0.04	140	0.56

B

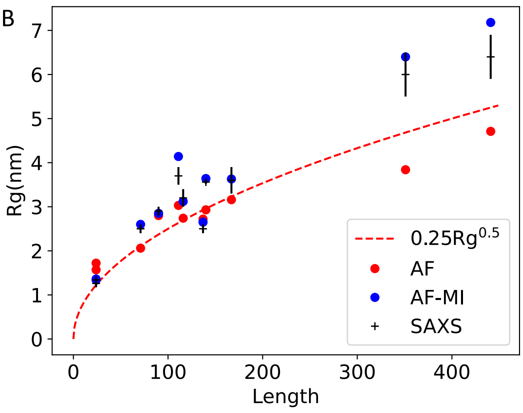

Supplementary Figure 4. Comparison of the values of the radius of gyration (Rg) from SAXS measurements with those from the AlphaFold (AF) and AlphaFold-Metainference (AF-MI) simulations. (A) Rg values predicted by AlphaFold-Metainference, AlphaFold individual structures and SAXS, as well as length and scaling exponent ν. **(B)** Rg as a function of protein length. We report predictions by AlphaFold-Metainference (blue), with AlphaFold individual structures (red) and SAXS data (black)[1]. The theoretical scaling of Rg with the length for a Flory random coil (ν=0.5) is shown as a dotted red line.

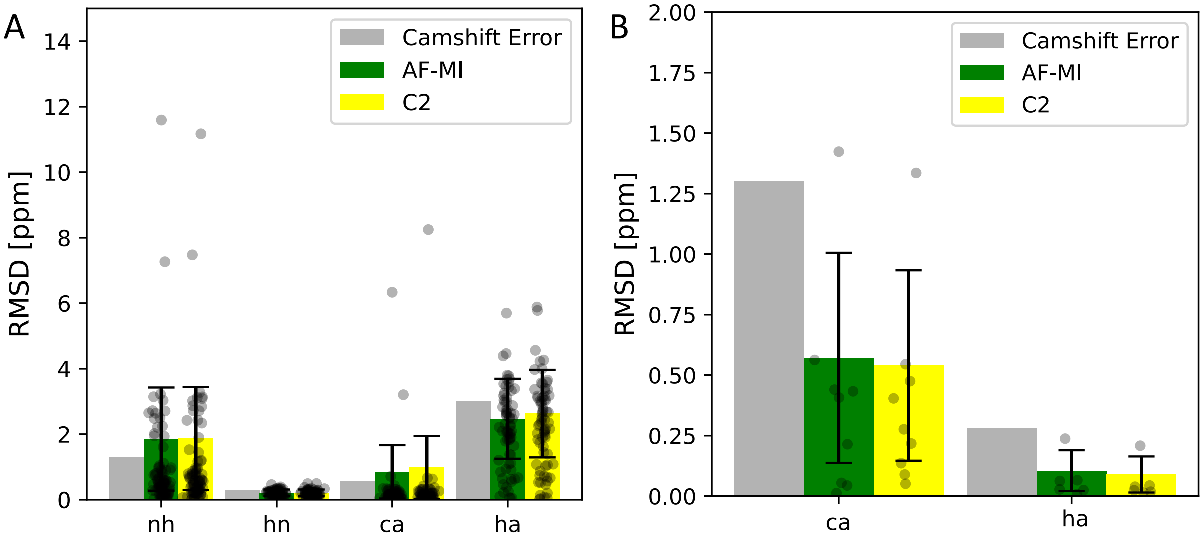

Supplementary Figure 5. Comparison of the back-calculated NMR chemical shifts from the CALVADOS-2 and AlphaFold-Metainference simulations. (A,B) Root mean ssquare deviation (RMSD) between CA, HA, HN, and NH chemicals shifts from Refs.[3,4] and predicted[5] ones from AlphaFold-Metainference (AF-MI, green) and CALVADOS-2 (C2) ensembles (yellow) for Sic1 (A) and AN16 (B)**.** The errors of the CamShift chemical shift predictions are shown as grey bars**.** In panel A, for each type of available chemical shift (NH, HN, CA, HA) the error bars represent the standard deviation of the RMSD of individual predicted chemical shifts in black dots from each experimental chemical shift datapoints (n_{NH}=71, n_{HN}=73, n_{CA}88, and n_{HA} =82). In panel B, since AN16 has 15 repeats of an 11-residue long peptide[4], the average and error bars deptict a block average and standard deviation through 15 repeats of either average CA or HA RMSD in black dots (n_{CA}=9 and n_{HA}=6) from the NMR chemical shift data per repeat.

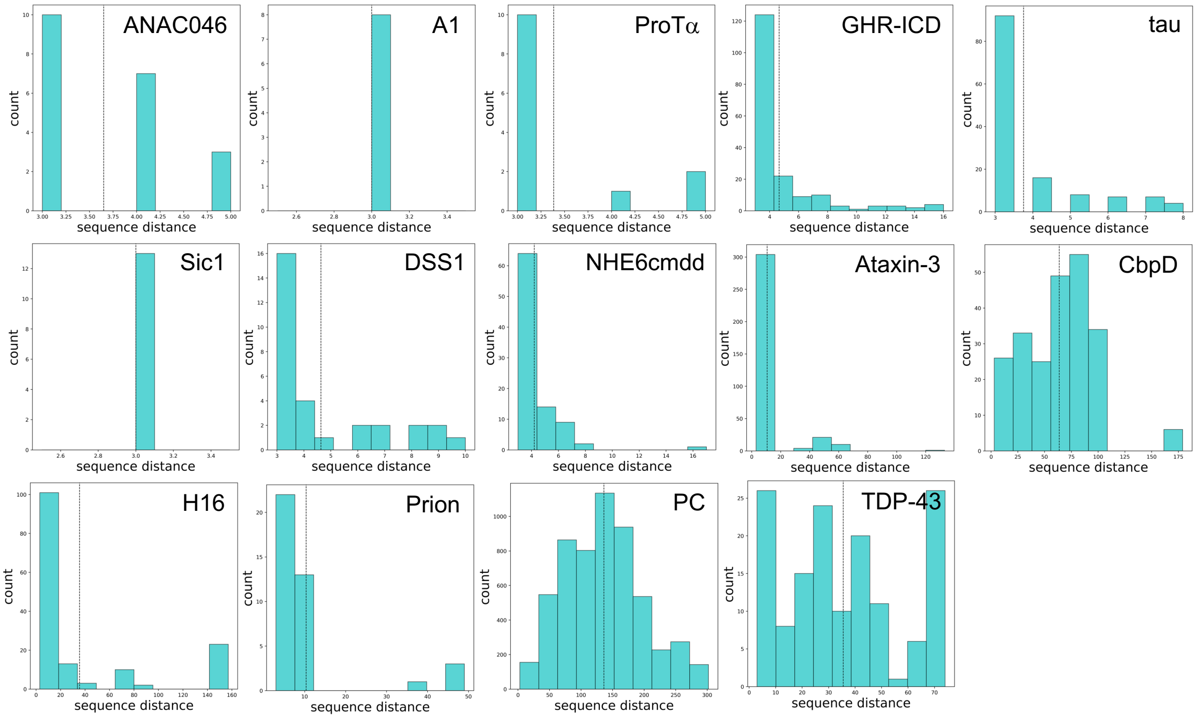

Supplementary Figure 6. Sequence separation of the pairwise distances used as structural restraints in the AlphaFold-Metainference simulations. The histograms show that distance along the sequence (i.e. the sequence separation) of residue pairs used as structural restraints of the highly disordered proteins shown in **Figures 1-2** tend to be small, while those of the partially disordered proteins shown in **Figures 3-6** can be quite large. The dotted lines indicate the average sequence separation of pairwise distances used as restraints per system.

A

	ΔRg/Rexp %						ΔRg/Rexp %
System	PAE<5	PAE<7	PAE<10	PAE<20	Hydropathy	Plddt>75 for >5 residues	Calvados
AN16	-15.60	-15.93	-18.74	-14.52	-0.98		-24
NLS	-35.83	-22.76	-22.92	-23.33	-2.28		-21
CTCF-RD	0.22	21.76	17.58	13.44	-0.73		15
Eralpha-NTD	9.85	42.23	41.30	12.67	-0.57		18
Msh6-NRT	-33.04	-9.63	-1.96	15.00	-1.417		-22
CortactinCRH	18.78	51.20	31.01	52.74	-1.2		14

B

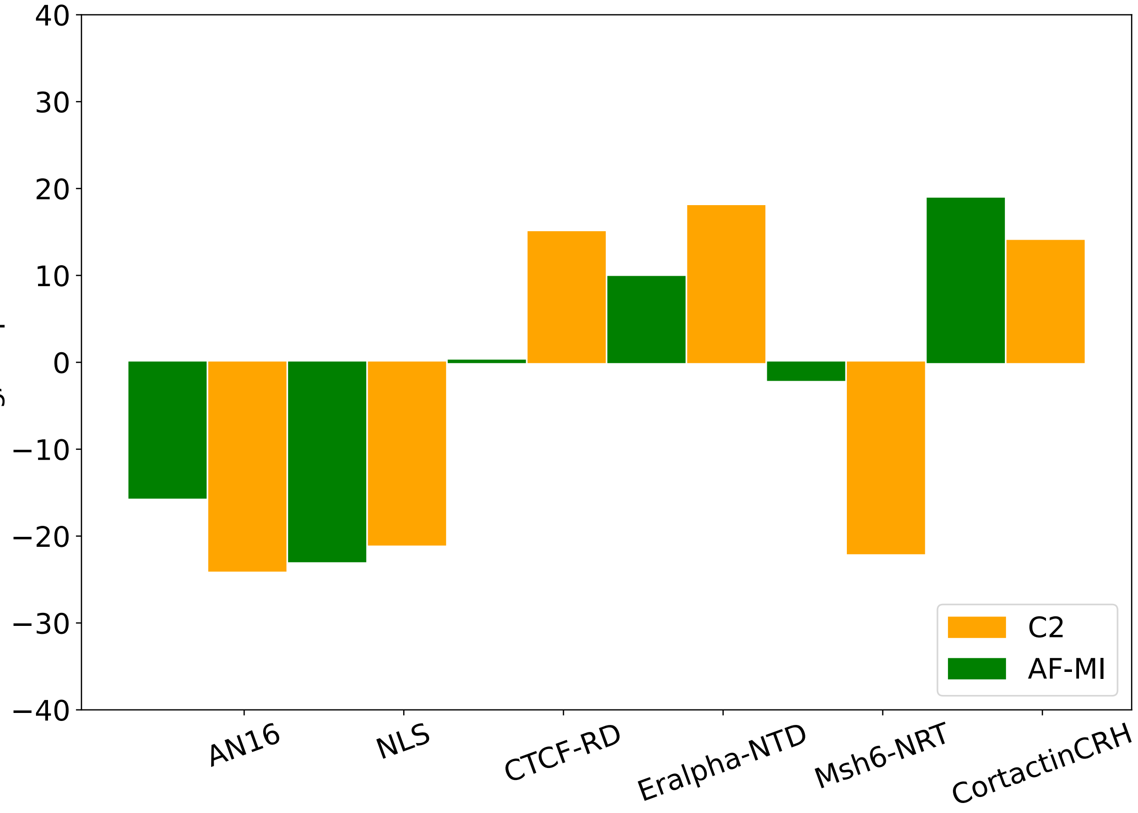

Supplementary Figure 7. Selection criterion for AlphaFold-predicted distances. (A) Comparison of the experimental values of the radius of gyration with those obtained from the AlphaFold-Metainference structural ensembles using different PAE values (<5, <7, <10, <20) for a set of benchmark proteins[5]. Proteins meeting the criterion of hydropathy < -1.4 and at least a 5-residue tract with pLDDT>75 are shown in green (PAE<10), while the remaining proteins are shown in red (PAE<5). **(B)** Comparison between the experimental values of the radius of gyration and those back-calculated from the AlphaFold-Metainference structural ensembles (green) and from previously reported CALVADOS-2 ensembles[5] (in orange). The values are obtained reported as block average and standard deviation between 3 independent samples obtained through resampling by Torrie Valleau metadynamics weights of the AlphaFold-Metainference ensemble, following a previously reported resampling procedure[6], using the distance selection criterion reported above.

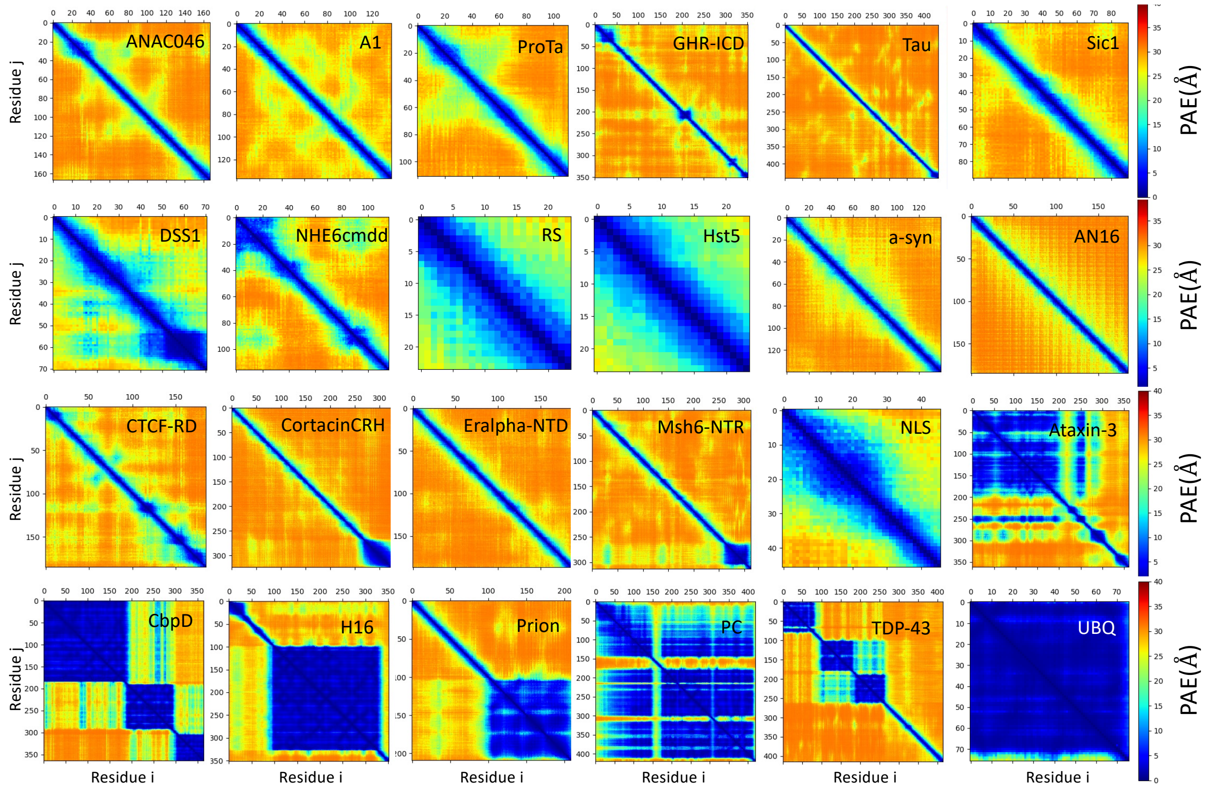

Supplementary Figure 8. AlphaFold PAE maps for the proteins analyzed in this work. The maps report the predicted aligned error (PAE) of AlphaFold for the intrinsically disordered proteins, partially disordered proteins and folded proteins that we used in this work.

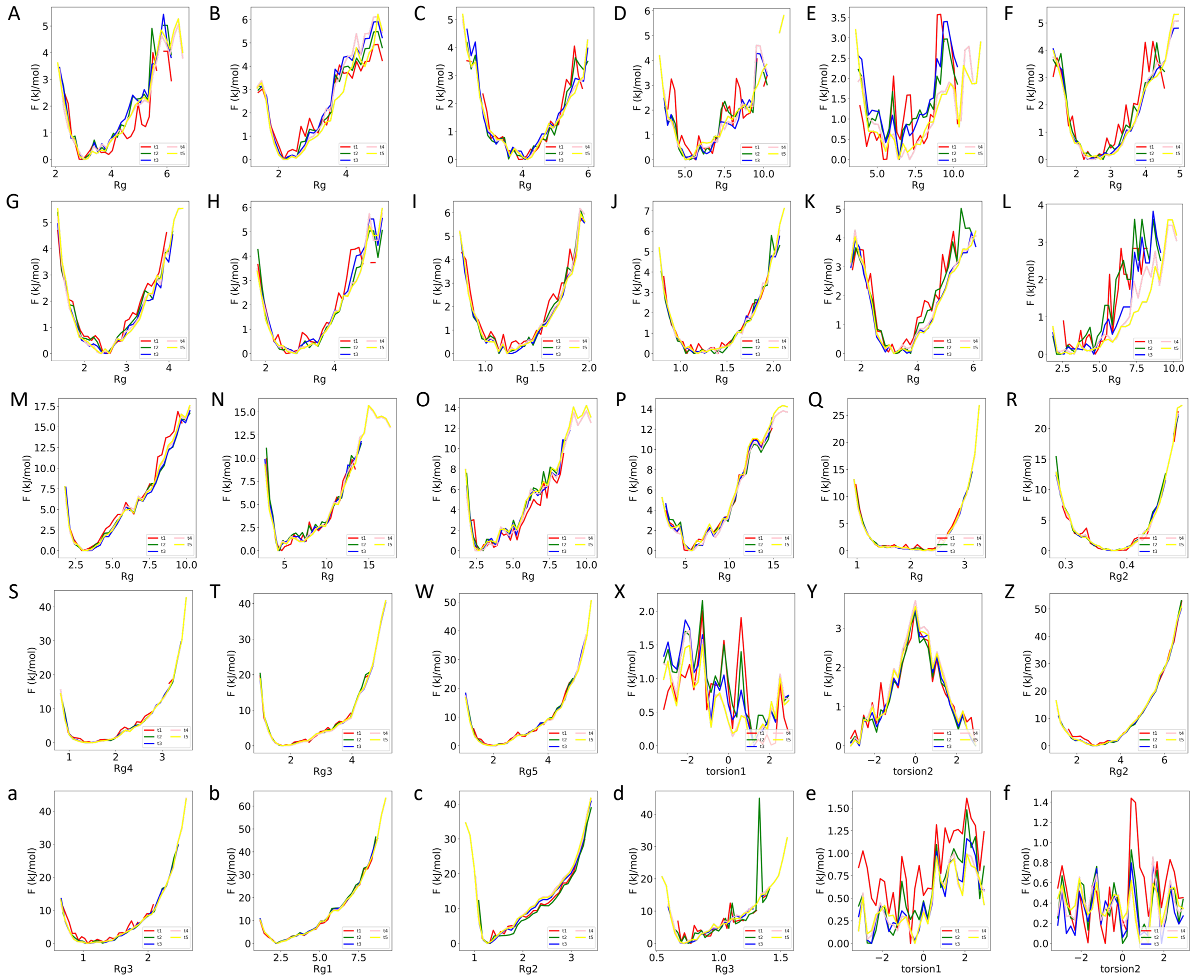

Supplementary Figure 9. Convergence analysis of the AlphaFold-Metainference simulations. Time-dependent free energy surfaces (kJ/mol), at 5 successive times (t1 to t5) along the biased collective variables for each protein (**Supplementary Table 1**): ANAC046 (A), A1 (B), ProTα (C), GHR-ICD (D), tau (E), Sic1 (F), DSS1 (G), NHE6cmdd (H), RS (I), Hst5 (J), α-synuclein (K), AN16 (L), CTCF-RD (M), CortactinCRH (N), Eralpha-NTD (O), Msh-NTR (P), NLS (Q), ataxin-3 (R-W), CbpD (X,Y), H16 (Z,a), prion (b), PC (c,d), TDP-43 (e,f).

System	Protein Length	Temp (K)	pH	Ionic strength (M)	Bias CV	Height (kJ/mol)	Bias Factor	Sequence
ANAC046	167	298	7.4	0.2	Rg	0.001	35	NAPSTTITTTKQLSRIDSLDNIDHL<br>LDFSSLPPLIDPGFLGQPGPSFSG<br>ARQQHDLKPVLHHPTTAPVDNTY<br>LPTQALNFPYHSVHNSGSDFGYG<br>AGSGNNNKGMIKLEHSLVSVSQE<br>TGLSSDVNTTATPEISSYPMMMN<br>PAMMDGSKSACDGLDDLIFWEDL<br>YTS
A1	137	298	7.4	0.2	Rg	0.001	35	GSMASASSSQRGRSGSGNFGGG<br>RGGGFGGNDNFGRGGNFSGRG<br>GFGGSRGGGGYGGSGDGYNGF<br>GNDGSNFGGGGSYNDFGNYNNQ<br>SSNFGPMKGGNFGGRSSGGSGG<br>GGQYFAKPRNQGGYGGSSSSSS<br>YGSGRRF
ProTa	111	298	7.4	0.2	Rg	0.001	35	GPSDAAVDTSSEITTKDLKEKKEV<br>VEEAENGRDAPANGNANEENGE<br>QEADNEVDEEEEEGGEEEEEEEE<br>GDGEEEDGDEDEEAESATGKRA<br>AEDDEDDDVDTKKQKTDEDD
GHR-ICD	351	298	7.4	0.2	Rg	0.001	35	SKQQRIKMLILPPVPVPKIKGIDPD<br>LLKEGKLEEVNTILAIHDSYKPEFH<br>SDDSWVEFIELDIDEPDEKTEESD<br>TDRLLSSDHEKSHSNLGVKDGDS<br>GRTSCCEPDILETDFNANDIHEGT<br>SEVAQPQRLKGEADLLCLDQKNQ<br>NNSPYHDACPATQQPSVIQAEKN<br>KPQPLPTEGAESTHQAAHIQLSNP<br>SSLSNIDFYAQVSDITPAGSVVLS<br>PGQKNKAGMSQCDMHPEMVSLC<br>QENFLMDNAYFCEADAKKCIPVA<br>PHIKVESHIQPSLNQEDIYITTESLT<br>TAAGRPGTGEHVPGSEMPVPDY<br>TSIHIVQSPQGLILNATALPLPDKE<br>FLSSCGYVSTDQLNKIMP
Tau	441	298	7.4	0.2	Rg	0.001	35	MAEPRQEFEVMEDHAGTYGLGD<br>RKDQGGYTMHQDQEGDTDAGLK<br>ESPLQTPTEDGSEEPGSETSDAK<br>STPTAEDVTAPLVDEGAPGKQAA<br>AQPHTEIPEGTTAEEAGIGDTPSL<br>EDEAAGHVTQARMVSKSKDGTG<br>SDDKKAKGADGKTKIATPRGAAP<br>PGQKGQANATRIPAKTPPAPKTP<br>PSSGEPPKSGDRSGYSSPGSPG<br>TPGSRSRTPSLPTPPTREPKKVA<br>VVRTPPKSPSSAKSRLQTAPVPM<br>PDLKNVKSKIGSTENLKHQPGGG<br>KVQIINKKLDLSNVQSKCGSKDNI<br>KHVPGGGSVQIVYKPVDLSKVTS<br>KCGSLGNIHHKPGGGQVEVKSEK<br>LDFKDRVQSKIGSLDNITHVPGGG<br>NKKIETHKLTFRENAKAKTDHGAE<br>IVYKSPVVSGDTSPRHLSNVSSTG<br>SIDMVDSPQLATLADEVSASLAKQ<br>GL
Sic1	90	298	7.4	0.2	Rg	0.001	35	MTPSTPPRSRGTRYLAQPSGNTS<br>SSALMQGQKTPQKPSQNLVPVTP<br>STTKSFKNAPLLAPPNSNMGMTS<br>PFNGLTSPQRSPFPKSSVKRT
DSS1	71	298	7.4	0.2	Rg	0.001	35	MSRAALPSLENLEDDDEFEDFAT<br>ENWPMKDTELDTGDDTLWENNW<br>DDEDIGDDDFSVQLQAELKKKGV<br>AAC
NHE6cmdd	116	298	7.4	0.2	Rg	0.001	35	GPPLTTTLPACCGPIARCLTSPQA<br>YENQEQLKDDDSDLILNDGDISLT<br>YGDSTVNTEPATSSAPRRFMGNS<br>SEDALDRELAFGDHELVIRGTRLV<br>LPMDDSEPPLNLLDNTRHGPA
RS	24	298	7.4	0.2	Rg	0.001	35	GAMGPSYGRSRSRSRSRSRSRS<br>RS
Hst5	24	298	7.4	0.2	Rg	0.001	35	DSHAKRHHGYKRKFHEKHHSHR<br>GY
a-synuclein	140	298	7.4	0.2	Rg	0.001	35	MDVFMKGLSKAKEGVVAAAEKTK<br>QGVAEAAGKTKEGVLYVGSKTKE<br>GVVHGVATVAEKTKEQVTNVGGA<br>VVTGVTAVAQKTVEGAGSIAAAT<br>GFVKKDQLGKNEEGAPQEGILED<br>MPVDPDNEAYEMPSEEGYQDYE<br>PEA
AN16	185	293	7	0.15	Rg	0.1	10	MHHHHHHPGAPAQTPSSQYGAP<br>AQTPSSQYGAPAQTPSSQYGAPA<br>QTPSSQYGAPAQTPSSQYGAPA<br>QTPSSQYGAPAQTPSSQYGAPA<br>QTPSSQYGAPAQTPSSQYGAPA<br>QTPSSQYGAPAQTPSSQYGAPA<br>QTPSSQYGAPAQTPSSQYGAPA<br>QTPSSQYGAPAQTPSSQYGAPA<br>QTPSSQYV
NLS	46	296	7.4	0.15	Rg	0.1	10	ACETNKRKREQISTDNEAKMQIQ<br>EEKSPKKKRKKRSSKANKPPECA

CTCF-RD	185	283	8	0.28	Rg	0.1	10	SAERRNSILTETLHRFSLEGDAPV<br>SWTETKKQSFKQTGEFGEKRKNS<br>ILNPINSIRKFSIVQKTPLQMNGIEE<br>DSDEPLERRLSLVPDSEQGEAILP<br>RISVISTGPTLQARRRQSVLNLMT<br>HSVNQGQNIHRKTTASTRKVSLA<br>PQANLTELDIYSRRLSQETGLEISE<br>EINEEDLKECFFDDME
Eralpha-NTD	187	283	7.4	0.1	Rg	0.1	10	SNAMTMTLHTKASGMALLHQIQG<br>NELEPLNRPQLKIPLERPLGEVYL<br>DSSKPAVYNYPEGAAYEFNAAAA<br>ANAQVYGQTGLPYGPGSEAAAF<br>GSNGLGGFPPLNSVSPSPLMLLH<br>PPPQLSPFLQPHGQQVPYYLENE<br>PSGYTVREAGPPAFYRPNSDNRR<br>QGGRERLASTNDKGSMAMESAK<br>ETRY
Msh6-NRT	313	293	8	0.12	Rg	0.1	10	MAPATPKTSKTAHFENGSTSSQK<br>KMKQSSLLSFFSKQVPSGTPSKK<br>VQKPTPATLENTATDKITKNPQGG<br>KTGKLFVDVDEDNDLTIAEETVST<br>VRSDIMHSQEPQSDTMLNSNTTE<br>PKSTTTDEDLSSSQSRRNHKRRV<br>NYAESDDDDSDTTFTAKRKKGKV<br>VDSESDEDEYLPDKNDGDEDDDI<br>ADDKEDIKGELAEDSGDDDDLISL<br>AETTSKKKFSYNTSHSSSPFTRNI<br>SRDNSKKKSRPNQAPSRSYNPSH<br>SQPSATSKSSKFNKQNEERYQWL<br>VDERDAQRRPKSDPEYDPRTLYI<br>PALEHHHHHH
CortactinCRH	324	295	8	0.31	Rg	0.1	10	GPLGSGYGGKFGVEQDRMDKSA<br>VGHEYQSKLSKHCSQVDSVRGF<br>GGKFGVQMDRVDQSAVGFEYQG<br>KTEKHASQKDYSSGFGGKYGVQ<br>ADRVDKSAVGFDYQGKTEKHES<br>QRDYSKGFGGKYGIDKDKVDKSA<br>VGFEYQGKTEKHESQKDYVKGF<br>GGKFGVQTDRQDKCALGWDHQE<br>KLQLHESQKDYKTGFGGKFGVQS<br>ERQDSAAVGFDYKEKLAKHESQQ<br>DYSKGFGGKYGVQKDRMDKNAS<br>TFEDVTQVSSAYQKTVPVEAVTS<br>KTSNIRANFENLAKEKEQEDRRKA<br>EAERAQRMAKERQEQEEARRKL<br>EEQARAKTQT
Ataxin-3	361	298	7.4	0.2	Rg2(184-187),<br>Rg3(192-246),<br>Rg4(256-290),<br>Rg5(302-361)	0.5	35	MESIFHEKQEGSLCAQHCLNNLL<br>QGEYFSPVELSSIAHQLDEEERM<br>RMAEGGVTSEDYRTFLQQPSGN<br>MDDSGFFSIQVISNALKVWGLELIL<br>FNSPEYQRLRIDPINERSFICNYKE<br>HWFTVRKLGKQWFNLNSLLTGPE<br>LISDTYLALFLAQLQQEGYSIFVVK<br>GDLPDCEADQLLQMIRVQQMHRP<br>KLIGEELAQLKEQRVHKTDLERVL<br>EANDGSGMLDEDEEDLQRALALS<br>RQEIDMEDEEADLRRAIQLSMQG<br>SSRNISQDMTQTSGTNLTSEELR<br>KRREAYFEKQQQKQQQQQQQQ<br>QQGDLSGQSSHPCERPATSSGA<br>LGSDLGDAMSEEDMLQAAVTMSL<br>ETVRNDLKTEGKK
CbpD	364	298	7.4	0.2	torsion1[1-<br>92,93-<br>184,192-<br>240,241-290],<br>torsion2[192-<br>240,241-<br>290,306-<br>334,335-364]	0.5	35	HGSMETPPSRVYGCFLEGPENPK<br>SAACKAAVAAGGTQALYDWNGV<br>NQGNANGNHQAVVPDGQLCGAG<br>KALFKGLNLARSDWPSTAIAPDAS<br>GNFQFVYKASAPHATRYFDFYITK<br>DGYNPEKPLAWSDLEPAPFCSITS<br>VKLENGTYRMNCPLPQGKTGKHV<br>IYNVWQRSDSPEAFYACIDVSFSG<br>AVANPWQALGNLRAQQDLPAGA<br>TVTLRLFDAQGRDAQRHSLTLAQ<br>GANGAKQWPLALAQKVNQDSTL<br>VNIGVLDAYGAVSPVASSQDNQV<br>YVRQAGYRFQVDIELPVEGGGEQ<br>PGGDGKVDFDYPQGLQQYDAGT<br>VVRGADGKRYQCKPYPNSGWCK<br>GWDLYYAPGKGMAWQDAWTLL
H16	351	298	7.4	0.2	Rg2 (28-99),<br>Rg3(328-351)	0.5	35	MATLEKLMKAFESLKSFQQQQQQ<br>QQQQQQQQQQPPPPPPPPPPPQ<br>LPQPPPQAQPLLPQPQPPPPPPP<br>PPPGPAVAEEPLHRPEASLEVLF<br>QGPGSHMASKGEELFTGVVPILV<br>ELDGDVNGHKFSVRGEGEGDAT<br>NGKLTLKFICTTGKLPVPWPTLVT<br>TLTYGVQCFSRYPDHMKRHDFFK<br>SAMPEGYVQERTISFKDDGTYKT<br>RAEVKFEGDTLVNRIELKGIDFKE<br>DGNILGHKLEYNFNSHNVYITADK<br>QKNGIKANFKIRHNVEDGSVQLAD<br>HYQQNTPIGDGPVLLPDNHYLST<br>QSVLSKDPNEKRDHMVLLEFVTA<br>AGITHGMDELYKLERAPGGGSHH<br>HHHH

Prion	209	298	7.4	0.2	Rg1(1-105)	0.5	35	KKRPKPGGWNTGGSRYPGQGSP<br>GGNRYPPQGGGGWGQPHGGG<br>WGQPHGGGWGQPHGGGWGQP<br>HGGGWGQGGGTHSQWNKPSKP<br>KTNMKHMAGAAAAGAVVGGLGG<br>YMLGSAMSRPIIHFGSDYEDRYY<br>RENMHRYPNQVYYRPMDEYSNQ<br>NNFVHDCVNITIKQHTVTTTTKGE<br>NFTETDVKMMERVVEQMCITQYE<br>RESQAYYQRGSS
PC	419	298	7.4	0.2	Rg2(146-179),<br>Rg3(302-315)	0.5	35	ANSFLEELRHSSLERECIEEICDFE<br>EAKEIFQNVDDTLAFWSKHVDGD<br>QCLVLPLEHPCASLCCGHGTCID<br>GIGSFSCDCRSGWEGRFCQREV<br>SFLNCSLDNGGCTHYCLEEVGW<br>RRCSCAPGYKLGDDLLQCHPAVK<br>FPCGRPWKRMEKKRSHLKRDTE<br>DQEDQVDPRLIDGKMTRRGDSP<br>WQVVLLDSKKKLACGAVLIHPSW<br>VLTAAHCMDESKKLLVRLGEYDL<br>RRWEKWELDLDIKEVFVHPNYSK<br>STTDNDIALLHLAQPATLSQTIVPI<br>CLPDSGLAERELNQAGQETLVTG<br>WGYHSSREKEAKRNRTFVLNFIKI<br>PVVPHNECSEVMSNMVSENMLC<br>AGILGDRQDACEGDSGGPMVASF<br>HGTWFLVGLVSWGEGCGLLHNY<br>GVYTKVSRYLDWIHGHIRDKEAP<br>QKSWAP
TDP-43	414	298	7.4	0.2	torsion1[3-<br>40,41-79,104-<br>140,141-178],<br>torsion2[104-<br>140,141-<br>178,191-<br>225,226-260]	0.5	35	EIPSEDDGTVLLSTVTAQFPGACG<br>LRYRNPVSQCMRGVRLVEGILHA<br>PDAGAGNLVYVVNYPKDNKRKM<br>DETDASSAVKVKRAVQKTSDLIVL<br>GLPAKTTEQDLKEYFSTFGEVLM<br>VQVKKDLKTGHSKGFGFVRFTEY<br>ETQVKVMSQRHMIDGRACDCKLP<br>NSKQSQDEPLRSRKVFVGRCTED<br>MTEDELREFFSQYGDVMDVFIPK<br>PFRAFAFVTFADDQIAQSLCGEDL<br>IIKGISVHISNAEPKHNSNRQLERS<br>GRFGGNPGGFGNQGGFGNSRG<br>GGAGLGNNQGSNMGGGMNFGA<br>FSINPAMMAAAQAALQSSAGMM<br>GMLASQQNQSGPSGNNQNQGN<br>MQREPNQAFGSGNNSYSGSNSG<br>AAIGAGSASNAGSGSGFNGGFGS<br>SMDSKSSGAGM

Supplementary Table 1. AlphaFold-Metainference simulations. For all the proteins studied in this work, we report the length, temperature (Kelvin), pH, ionic strength (molar), biased CV, metadynamics height (kJ/mol), and bias factor.

Supplementary References

1 Pesce, F. *et al.* Assessment of models for calculating the hydrodynamic radius of intrinsically disordered proteins. *Bioph. J.* **122**, 310-321 (2023).

2 Gomes, G.-N. W. *et al.* Conformational ensembles of an intrinsically disordered protein consistent with NMR, saxs, and single-molecule fret. *J. Am. Chem. Soc.* **142**, 15697-15710 (2020).

3 Nairn, K. M. *et al.* A synthetic resilin is largely unstructured. *Bioph. J.* **95**, 3358-3365 (2008).

4 Robustelli, P., Kohlhoff, K., Cavalli, A. & Vendruscolo, M. Using NMR chemical shifts as structural restraints in molecular dynamics simulations of proteins. *Structure* **18**, 923-933 (2010).

5 Tesei, G. *et al.* Conformational ensembles of the human intrinsically disordered proteome. *Nature* **626**, 897-904 (2024).

6 Löhr, T., Camilloni, C., Bonomi, M. & Vendruscolo, M. A practical guide to the simultaneous determination of protein structure and dynamics using metainference. *Biomolecular Simulations: Methods and Protocols*, 313-340 (2019).
