# Supplementary material for: AlphaFold prediction of structural ensembles of disordered proteins
Source: Nat Commun. 2025 Feb 14;16:1632. doi: 10.1038/s41467-025-56572-9 (PMC11829000; doi:10.1038/s41467-025-56572-9)
Supplement: Supplementary file 2 — Reporting Summary [file 41467_2025_56572_MOESM2_ESM.pdf]

nature portfolio

Corresponding author(s): Michele Vendruscolo

Last updated by author(s): 22 January 2025

Reporting Summary

Nature Portfolio wishes to improve the reproducibility of the work that we publish. This form provides structure for consistency and transparency in reporting. For further information on Nature Portfolio policies, see our Editorial Policies and the Editorial Policy Checklist.

Statistics

For all statistical analyses, confirm that the following items are present in the figure legend, table legend, main text, or Methods section.

n/a	Confirmed	
☐	☒	The exact sample size (*n*) for each experimental group/condition, given as a discrete number and unit of measurement
☐	☒	A statement on whether measurements were taken from distinct samples or whether the same sample was measured repeatedly
☒	☐	The statistical test(s) used AND whether they are one- or two-sided<br>*Only common tests should be described solely by name; describe more complex techniques in the Methods section.*
☐	☒	A description of all covariates tested
☐	☒	A description of any assumptions or corrections, such as tests of normality and adjustment for multiple comparisons
☐	☒	A full description of the statistical parameters including central tendency (e.g. means) or other basic estimates (e.g. regression coefficient) AND variation (e.g. standard deviation) or associated estimates of uncertainty (e.g. confidence intervals)
☒	☐	For null hypothesis testing, the test statistic (e.g. *F*, *t*, *r*) with confidence intervals, effect sizes, degrees of freedom and *P* value noted<br>*Give P values as exact values whenever suitable.*
☐	☒	For Bayesian analysis, information on the choice of priors and Markov chain Monte Carlo settings
☒	☐	For hierarchical and complex designs, identification of the appropriate level for tests and full reporting of outcomes
☒	☐	Estimates of effect sizes (e.g. Cohen's *d*, Pearson's *r*), indicating how they were calculated

Our web collection on statistics for biologists contains articles on many of the points above.

Software and code

Policy information about availability of computer code

Data collection

We used either existing openly available datasets (see data availability section) or generated the data using the following codes:
Available data:
a) 11 IDP CALVADOS2 ensembles: https://doi.org/10.5281/zenodo.14712644
b) 11 IDP SAXS data: https://github.com/KULL-Centre/papers/tree/main/2022/rh-fwd-model-pesce-et-al/SAXS
c) 6 PDP SAXS data were found in https://www.sasbdb.org (SAS IDs in manuscript)

Code:
a) Custom code https://github.com/vendruscolo-lab/AlphaFold-IDP, able to setup and generate structural ensembles by using AlphaFold2 distances as restraints (example provided). This custom code involves
a1) Custom alphafold2 distance prediction notebook: https://colab.research.google.com/github/zshengyu14/colabfold_distmat/blob/main/AlphaFold2.ipynb
a2) Custom, new openmm-plumed interface: https://github.com/vendruscolo-lab/OpenMM-Plumed-MPI
a3) PLUMED-v2.8.0 package used to perform metainference using AlphaFold2 distance restraints.
b) PULCHRA package https://cssb.biology.gatech.edu/skolnick/files/PULCHRA/index.html was utilized to backmap the coarse-grained structural ensemble to the atomistic

Generated data:
All thenerated AlphaFold-Metainference ensembles can be found in https://zenodo.org/records/7756138#.ZBnysy0Rq1E (AF_MIinput.zip,AF_MIensembles.zip)

Data analysis	python3.9, matplotlib-v3.5.2, pandas-v1.4.4,numpy-v1.21.5, mdtraj-v1.9.7, jupyter-notebook-v6.4.12, plumed-v2.8.0dev, chimerax-v1.2.5, Raw-v2.1.4

For manuscripts utilizing custom algorithms or software that are central to the research but not yet described in published literature, software must be made available to editors and reviewers. We strongly encourage code deposition in a community repository (e.g. GitHub). See the Nature Portfolio guidelines for submitting code & software for further information.

Data

Policy information about availability of data

All manuscripts must include a data availability statement. This statement should provide the following information, where applicable:

- Accession codes, unique identifiers, or web links for publicly available datasets
- A description of any restrictions on data availability
- For clinical datasets or third party data, please ensure that the statement adheres to our policy

AlphaFold Metainference ensemble generation procedure, openmm-plumed interface, Alpha-Fold pairwise aminoacid distance predictor google collab, back mapping and comparison to the experimental SAXS based pair distance distribution function code developed in the paper have been deposited in the public repository, https://github.com/vendruscolo-lab/AlphaFold-IDP. All the AlphaFold Metainference generated ensembles of this study have been deposited in the Zenodo repository, https://doi.org/10.5281/zenodo.14712644

Human research participants

Policy information about studies involving human research participants and Sex and Gender in Research.

Reporting on sex and gender	N/A
Population characteristics	N/A
Recruitment	N/A
Ethics oversight	N/A

Note that full information on the approval of the study protocol must also be provided in the manuscript.

Field-specific reporting

Please select the one below that is the best fit for your research. If you are not sure, read the appropriate sections before making your selection.

☒ Life sciences ☐ Behavioural & social sciences ☐ Ecological, evolutionary & environmental sciences

For a reference copy of the document with all sections, see nature.com/documents/nr-reporting-summary-flat.pdf

Life sciences study design

All studies must disclose on these points even when the disclosure is negative.

Sample size	The sample size of our AlphaFold Metainference structural ensembles was chosen so that it represented a converged structural ensemble. In other words the equilibrium properties did not change when we further extended the sampling.
Data exclusions	No data were excluded
Replication	For restraining to the AlphaFold experimental distance using Metainference and CALVADOS-2, no replication was necessary as Metainference already deals with the multiple copies of the system. In particular each AlphaFold-Metainference simulation involved six replicas which run parallel and the AF distance restraints act on the average over the 6 replicas
Randomization	The six replicas in AlphaFold-Metainference start from different initial configurations obtained from a short nvt equilibration. The velocities of these initial configurations are randomized.
Blinding	No intervention was performed by the investigators in the generation of the structural ensembles by AlphaFold-Metainference method. The meth method generated the ensembles and a meta-analysis was perfomed on it to compare to experimental SAXS data, as described in the manuscript

Reporting for specific materials, systems and methods

We require information from authors about some types of materials, experimental systems and methods used in many studies. Here, indicate whether each material, system or method listed is relevant to your study. If you are not sure if a list item applies to your research, read the appropriate section before selecting a response.

Materials & experimental systems

n/a	Involved in the study
☒	☐ Antibodies
☒	☐ Eukaryotic cell lines
☒	☐ Palaeontology and archaeology
☒	☐ Animals and other organisms
☒	☐ Clinical data
☒	☐ Dual use research of concern

Methods

n/a	Involved in the study
☒	☐ ChIP-seq
☒	☐ Flow cytometry
☒	☐ MRI-based neuroimaging
